# Integrated psychiatric and surgical care in managing self-inflicted abdominal trauma: a case report in a resource-limited setting

**DOI:** 10.1097/MS9.0000000000002593

**Published:** 2024-09-23

**Authors:** Pratik Adhikari

**Affiliations:** B.P. Koirala Institute of Health Sciences, Dharan, Nepal

**Keywords:** alcohol dependence, integrated psychiatric and surgical care, schizophrenia, self-inflicted abdominal trauma

## Abstract

**Introduction and importance:**

Self-inflicted abdominal stab wounds present rare yet critical challenges requiring urgent surgical intervention and comprehensive psychiatric evaluation. Such injuries often correlate with psychiatric disorders like schizophrenia and alcohol dependence, posing significant clinical and psychological management hurdles.

**Case presentation:**

A 36-year-old male with a history of chronic alcohol use and schizophrenia presented with a self-inflicted stab wound to the abdomen following auditory hallucinations. Upon admission, he was hemodynamically unstable, with eviscerated bowel loops and a lacerated greater omentum. Prompt surgical exploration revealed extensive abdominal trauma necessitating meticulous repair and intensive postoperative care.

**Clinical discussion:**

The case highlights the intricate interplay between severe psychiatric illness and self-harm, exacerbated by alcohol intoxication. Integrated psychiatric and surgical care proved pivotal in achieving stabilization and facilitating recovery. The challenges encountered in a resource-limited setting included a lack of advanced imaging modalities, limited access to specialized psychiatric care, and constraints in postoperative monitoring equipment, underscoring the importance of adaptive management strategies.

**Conclusion:**

This report emphasizes the critical need for a multidisciplinary approach in managing self-inflicted abdominal injuries. The successful outcome underscores the efficacy of timely surgical intervention and comprehensive psychiatric support. Integrated care models are essential for addressing the complex needs of patients with psychiatric comorbidities, ensuring holistic management, and reducing the risk of recurrence in similar cases.

## Introduction

Highlights

- Integration of timely surgical intervention and psychiatric evaluation stabilized a patient with severe self-inflicted abdominal trauma.
- The case underscores the critical interplay between schizophrenia, alcohol dependence, and self-harm behaviors.
- Challenges in a resource-limited setting necessitated adaptive management strategies for comprehensive care.
- Successful outcomes demonstrate the effectiveness of integrated psychiatric and surgical approaches in complex trauma cases.

Self-inflicted abdominal stab wounds are a relatively rare but severe form of self-harm that often requires immediate surgical intervention and comprehensive psychiatric evaluation. Such injuries present significant challenges in clinical management and psychological assessment. The incidence of self-inflicted abdominal injuries has been associated with various psychiatric disorders, including schizophrenia, major depressive disorder, and substance abuse, particularly alcohol dependence^1^. Recent studies have shown an increasing trend in self-inflicted injuries globally, necessitating a multidisciplinary approach to treatment that addresses both the physical and mental health aspects of the patient^2^.

Alcohol consumption and chronic psychiatric illnesses, such as schizophrenia, are major risk factors for self-inflicted injuries. Schizophrenia, particularly when accompanied by auditory hallucinations, can significantly impair judgment and increase the risk of self-harm. Auditory hallucinations have been identified as a critical factor in the impulsivity and compulsion that lead to self-inflicted injuries^3^. This case highlights the complex interplay between chronic alcohol use and schizophrenia, which culminated in a severe self-inflicted abdominal injury. Understanding the underlying psychiatric conditions and their manifestations is crucial for effectively managing such cases.

Managing self-inflicted abdominal injuries involves not only addressing the immediate physical trauma but also providing long-term psychiatric care to prevent recurrence. The integration of surgical and psychiatric care is essential in ensuring holistic recovery for patients with self-inflicted injuries^4^. This case report illustrates the importance of timely surgical intervention, thorough psychiatric evaluation, and the challenges encountered in resource-limited settings. The findings underscore the need for improved strategies to manage similar cases, including better psychiatric support and substance abuse rehabilitation programs.

## Case presentation

A 36-year-old male presented to the emergency room with a self-inflicted stab wound to the abdomen using a khukuri (a traditional Nepali knife). The patient experienced loss of consciousness immediately following the injury. There was no significant family history of psychiatric or medical illnesses. The patient has a known history of chronic alcohol use and smoking and is under medication for schizophrenia, specifically for auditory hallucinations.

The patient was reportedly well until the day of the incident. Under the influence of alcohol, he stabbed himself in the abdomen following auditory hallucinations that led to feelings of shame and hopelessness. The patient reported hearing the voices of three individuals, with one female voice repeatedly telling him he was being shamed on social media. This distress led him to attempt suicide by stabbing himself.

Upon arrival at the emergency department, the patient was in a state of hemodynamic instability. He had a Glasgow Coma Scale (GCS) score of 3/15, indicating severe impairment of consciousness. Given the absence of head trauma, the patient’s GCS of 3/15 was attributed to severe alcohol intoxication combined with shock from blood loss and psychological stress.

The patient was tachycardic with a heart rate of 100 beats per minute and a blood pressure of 120/70 mmHg. Oxygen saturation (SpO_2_) was 97%.

**Figure 1:**
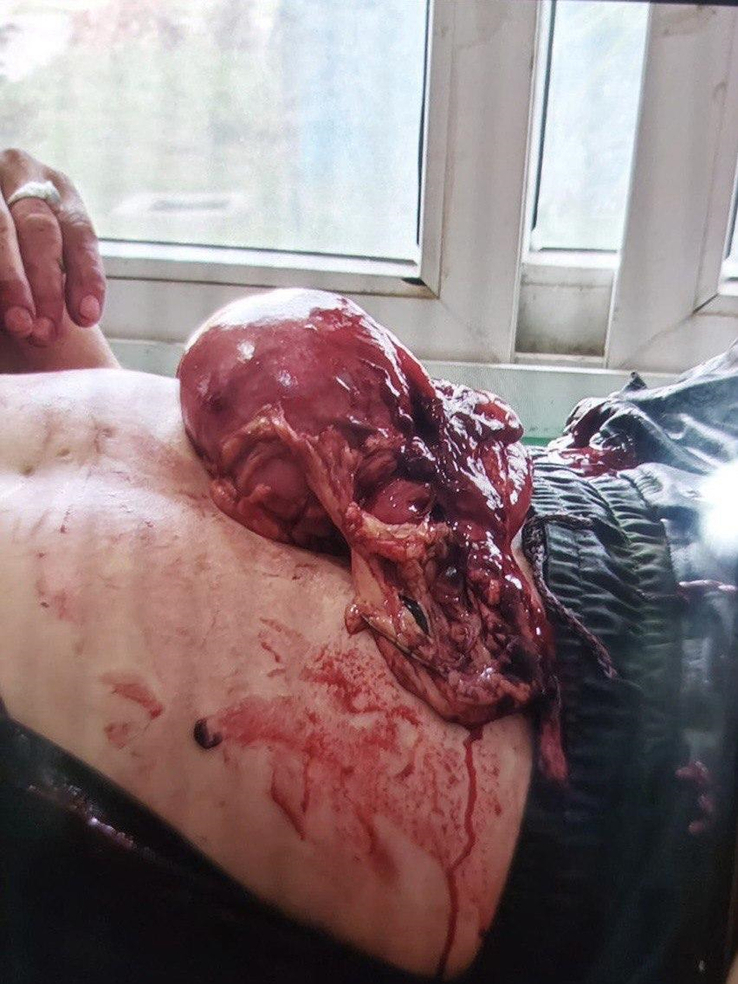
Shows the patient’s eviscerated bowel loops and stomach immediately following the self-inflicted abdominal stab wound. The protruding organs are exposed and resting on the patient’s abdomen.

Abdominal examination revealed eviscerated bowel loops with no active bleeding, as seen in the provided images. The wound measured ~10 cm in length, located 5 cm below and parallel to the left subcostal margin, with eviscerated bowel loops and stomach immediately following the self-inflicted abdominal stab wound. The protruding organs are exposed and resting on the patient’s abdomen, highlighting the severity of the injury before surgical intervention (Fig. 1).

Initial laboratory investigations revealed significant findings. The complete blood count showed a hemoglobin level of 13.5 gm/dl, a platelet count of 59 000 cells/mm³, and a total leukocyte count of 8500 cells/mm³. Liver function tests indicated elevated ALT (SGPT) levels at 273 U/l and AST (SGOT) at 457 U/l. The coagulation profile revealed a prothrombin time of 17 s, slightly above the normal range of 12–16 s. Other parameters were within normal limits except for mild hypokalemia, with a potassium level of 3.4 mmol/l. These findings are illustrated in Table 1. The low platelet count raised concerns about potential underlying liver disease or bone marrow suppression, which were investigated intraoperatively and postoperatively with no signs of cirrhosis or other abnormalities.

**Table 1:** Laboratory investigations

Test	Value	Normal range
Hemoglobin	13.5 gm/dl	13.0–17.0 gm/dl
Platelet count	59 000 cells/mm^3^	150 000–450 000 cells/mm^3^
Total leukocyte count	8500 cells/mm^3^	4000–110 000 cells/mm^3^
ALT (SGPT)	273 U/l	7–56 U/l
AST (SGOT)	456 U/l	10–40 U/l
Prothrombin time	17 s	12–16 s
Potassium (K)	3.4 mmol/l	3.5–5.1 mmol/l

Upon stabilization, the patient was taken to the operating room for an exploratory laparotomy under general anesthesia. Preoperative preparations included inserting an arterial line 16G and 18G cannulas and a central venous pressure (CVP) line under aseptic conditions. Anesthesia induction was achieved with fentanyl, propofol, and cisatracurium, followed by endotracheal intubation.

**Figure 2:**
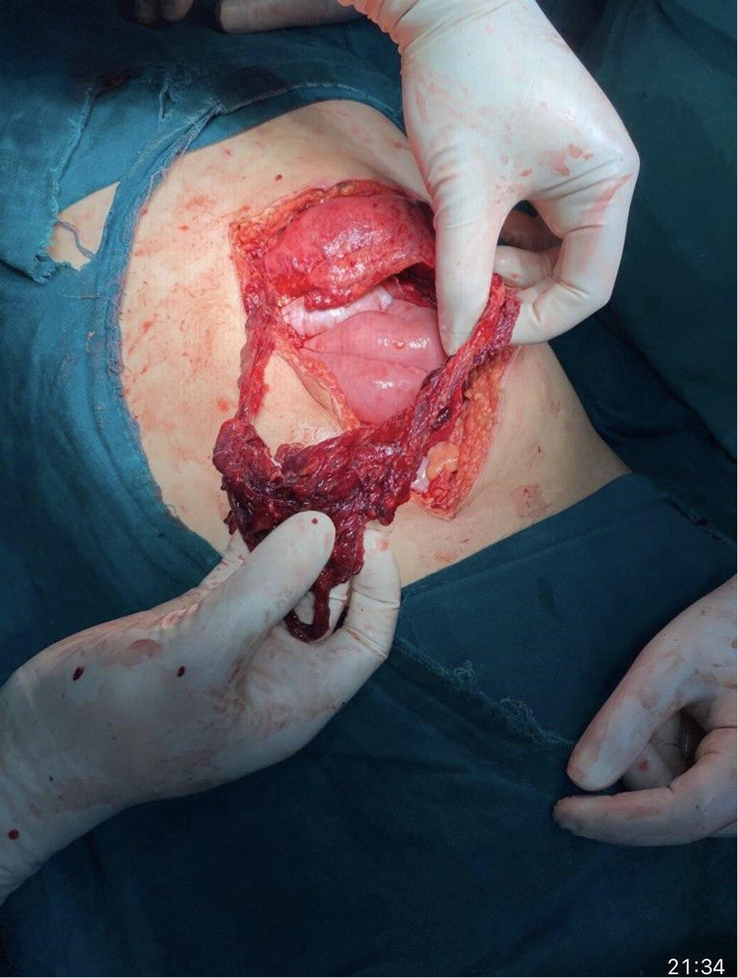
Shows the intraoperative finding of eviscerated bowel loops and the lacerated greater omentum being carefully examined by the surgical team. The extent of the abdominal injury is visible, with the omentum protruding through the abdominal incision.

**Figure 3:**
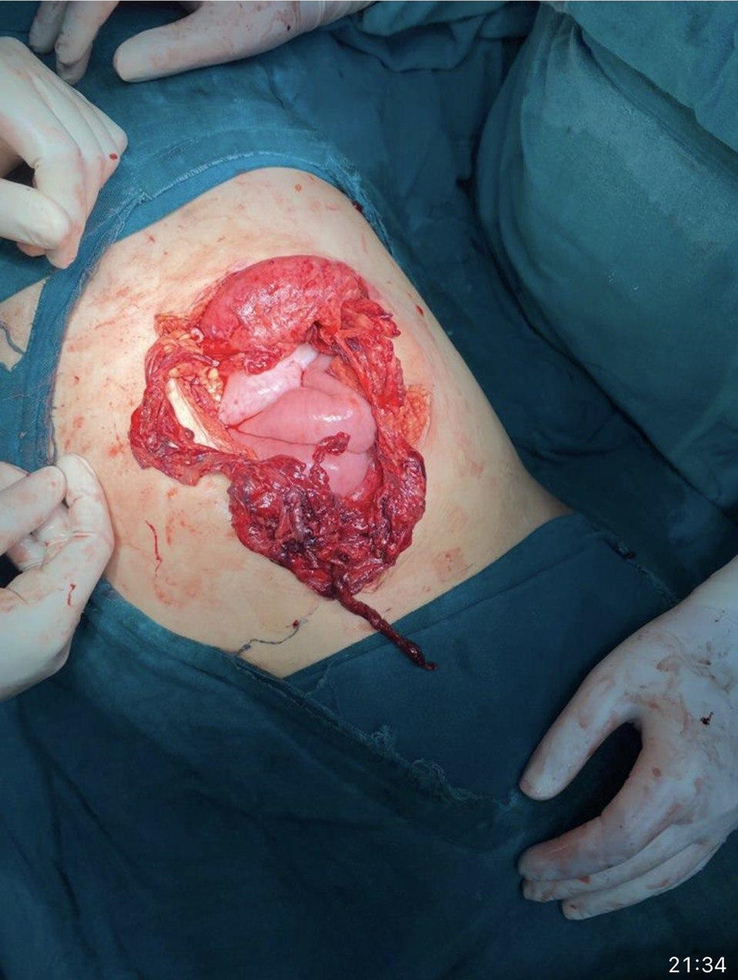
Shows a close-up of the surgical site with eviscerated bowel loops. The meticulous handling of the eviscerated organs and the extent of the abdominal trauma are evident, highlighting the severity of the injury and the need for precise surgical intervention.

Intraoperative findings confirmed a 10 cm cut injury with eviscerated stomach and bowel loops and a 10 cm laceration of the greater omentum (Figs. 2, 3). The ileum was edematous over a 15 cm length, 20 cm proximal to the ileocecal junction. The surgical team performed peritoneal lavage and resection of the lacerated greater omentum. Bowel loops were mobilized, and no additional bowel injury was noted. Hemostasis was achieved, and the abdominal cavity was closed in layers.

Postoperatively, the patient’s GCS improved to 10/15 within 12 h, indicating partial recovery of consciousness. The patient was monitored in the surgical intensive care unit (SICU) for hemodynamic stability and received intravenous antibiotics (Durataz), analgesics (thermodol, ketrol, and tramadol), antiemetics (omitron), and supportive care with intravenous fluids. Psychiatric evaluation confirmed the diagnosis of acute psychotic disorder (AIPD) with alcohol dependence syndrome (ADS). The patient received intravenous diazepam and thiamine-enriched fluids.

The patient was transferred to a special psychiatric ward after stabilization, where he received continuous psychiatric support and counseling to prevent a reattempt of suicide. He was enrolled in a long-term rehabilitation program for alcohol dependence and received regular psychiatric follow-ups to manage his schizophrenia and avoid recurrence.

## Limitations

This case underscores the significant challenges encountered when managing severe abdominal trauma within a resource-limited setting, where access to advanced medical technologies and specialized healthcare personnel may be restricted. Despite these constraints, the timely initiation of surgical intervention and meticulous perioperative management played crucial roles in stabilizing the patient and preventing further deterioration. The integration of psychiatric evaluation and care alongside surgical interventions was particularly essential in addressing the complex interplay between the patient’s severe psychiatric illness, including schizophrenia and alcohol dependence, and the self-inflicted nature of the trauma. This highlights the need for innovative approaches and adaptive strategies in delivering comprehensive, patient-centered care under challenging circumstances, aiming to optimize outcomes despite limited resources.

I am writing under the SCARE checklist. Under the SCARE 2023 guideline (Sohrabi *et al*., AQ5 2023), the methodology for reporting surgical case details was strictly adhered to in this study^5^.

## Discussion

Self-inflicted abdominal stab wounds present a unique set of challenges for healthcare providers. The immediate concern is stabilising the patient and addressing any life-threatening injuries through surgical intervention. In this case, the patient presented with eviscerated bowel loops and a lacerated greater omentum, requiring prompt surgical management^6^. The successful stabilization and surgical intervention in this case underscore the critical role of timely medical response in managing severe abdominal trauma^7^.

The psychiatric background of the patient, including a history of schizophrenia and alcohol dependence, highlights the complex interplay between mental health disorders and self-harm behaviors. Auditory hallucinations, particularly those involving derogatory or commanding voices, are a well-documented risk factor for self-inflicted injuries in patients with schizophrenia^8^. The patient’s hallucinations and the influence of alcohol likely exacerbated his impulsivity and contributed to the severity of the injury.

Addressing the psychiatric aspects of self-inflicted injuries is as important as managing the physical trauma. Comprehensive psychiatric evaluation and intervention are necessary to prevent recurrence and support long-term recovery. In this case, the patient was diagnosed with acute psychotic disorder and alcohol dependence syndrome, conditions that require ongoing psychiatric care and substance abuse treatment^9^. Integrating psychiatric and surgical care is essential for holistic management and recovery^10^.

Postoperative care in such cases is crucial for monitoring and managing potential complications. The patient’s postoperative period involved intensive care monitoring and administration of intravenous antibiotics, analgesics, and antiemetics. Such comprehensive care helps mitigate the risk of postoperative infections and ensure pain management, which are critical for recovery^11^.

The challenges faced in a resource-limited setting included limited access to advanced diagnostic tools, such as CT scans, which could have provided more detailed information about the extent of internal injuries. Additionally, there were constraints in accessing specialized psychiatric care and adequate postoperative monitoring equipment. These limitations necessitated adaptive management strategies, including reliance on clinical judgment for intraoperative decisions and leveraging available resources for psychiatric support^12^.

Despite these challenges, the multidisciplinary approach and timely intervention led to a successful outcome. The patient’s enrollment in a long-term rehabilitation program and continuous psychiatric support underscores the importance of comprehensive care in preventing recurrence and supporting recovery. This case highlights the need for improved strategies and resources in resource-limited settings to manage similar cases effectively^13^.

Psychiatric follow-up and support are crucial in preventing future self-harm incidents. The patient’s history of schizophrenia and alcohol dependence requires long-term psychiatric and psychological support to address underlying issues and avoid recurrence. Integrated care models that combine surgical, psychiatric, and substance abuse treatment are effective in managing such complex cases^14^.

## Conclusion

Self-inflicted abdominal stab wounds in patients with psychiatric disorders, particularly schizophrenia and alcohol dependence, pose significant clinical and psychological challenges. The successful management of this case underscores the importance of an integrated approach combining prompt surgical intervention with comprehensive psychiatric care. Adaptive strategies and multidisciplinary collaboration are essential for positive outcomes in resource-limited settings. Future strategies should focus on enhancing access to psychiatric care, improving substance abuse rehabilitation programs, and providing advanced diagnostic tools to support the effective management of such complex cases.

## Ethical approval

Since the study is a case-report, we did not obtain ethical approval.

## Consent

Written informed consent was obtained from the participant for publication and any accompanying images.

## Source of funding

Not applicable.

## Author contribution

P.A.: provided us with data and materials from the archive and their notes, wrote the manuscript, collected the images and put them in perspective according to the timeline of the case, reviewed the manuscript and did final editing, and read the final manuscript and approved the case.

## Conflicts of interest disclosure

Not applicable.

## Research registration unique identifying number (UIN)

This is a cross-sectional involving a human subject, so registration of the research study was done.

1. Registry used: Researchregistry.com.
2. Unique identifying number or registration ID: researchregistry10427.

## Guarantor

Pratik Adhikari is the guarantor of the study.

## Data availability statement

The datasets supporting the conclusions of this article are included within the article.

## Provenance and peer review

Not commissioned or externally peer-reviewed.
